# The evolution of early cellular systems viewed through the lens of biological interactions

**DOI:** 10.3389/fmicb.2015.01144

**Published:** 2015-10-19

**Authors:** Anthony M. Poole, Daniel Lundin, Kalle T. Rytkönen

**Affiliations:** ^1^Allan Wilson Centre for Molecular Ecology and Evolution, University of Canterbury, Christchurch, New Zealand; ^2^Biomolecular Interaction Centre, University of Canterbury, Christchurch, New Zealand; ^3^School of Biological Sciences, University of Canterbury, Christchurch, New Zealand; ^4^Department of Biochemistry and Biophysics, Arrhenius Laboratories, Stockholm University, Stockholm, Sweden; ^5^Centre for Ecology and Evolution in Microbial Model Systems, Linnaeus University, Kalmar, Sweden; ^6^Department of Ecology and Evolutionary Biology, Yale University, New Haven, CT, USA; ^7^Yale Systems Biology Institute, Yale University, New Haven, CT, USA

**Keywords:** minimal genome, distributed genome, last universal common ancestor, coevolution, evolutionary transitions, communal ancestor, levels of selection

## Abstract

The minimal cell concept represents a pragmatic approach to the question of how few genes are required to run a cell. This is a helpful way to build a parts-list, and has been more successful than attempts to deduce a minimal gene set for life by inferring the gene repertoire of the last universal common ancestor, as few genes trace back to this hypothetical ancestral state. However, the study of minimal cellular systems is the study of biological outliers where, by practical necessity, coevolutionary interactions are minimized or ignored. In this paper, we consider the biological context from which minimal genomes have been removed. For instance, some of the most reduced genomes are from endosymbionts and are the result of coevolutionary interactions with a host; few such organisms are “free-living.” As few, if any, biological systems exist in complete isolation, we expect that, as with modern life, early biological systems were part of an ecosystem, replete with organismal interactions. We favor refocusing discussions of the evolution of cellular systems on processes rather than gene counts. We therefore draw a distinction between a pragmatic minimal cell (an interesting engineering problem), a distributed genome (a system resulting from an evolutionary transition involving more than one cell) and the looser coevolutionary interactions that are ubiquitous in ecosystems. Finally, we consider the distributed genome and coevolutionary interactions between genomic entities in the context of early evolution.

## Introduction

The minimal genome concept is a theoretical idea that has been considered in two different arenas. One is synthetic biology, an area of biological engineering where there is interest in establishing the minimal machinery for a cell (Peterson and Fraser, 2001; Dewall and Cheng, 2011; Juhas et al., 2011; Acevedo-Rocha et al., 2013). The other is in cell origins, where there has been interest in establishing the nature of early cellular systems (Mushegian, 1999; Koonin, 2003). Synthetic biology has made strong technical advances, with key proof-of-principle results such as systematically screening for essential genes (Hutchison et al., 1999; Glass et al., 2006), synthetic genome assembly (Gibson et al., 2008), transformation of a cell with a chemical genome (Gibson et al., 2010), and the development of computational cellular models derived from genomic knowledge (Karr et al., 2012).

The synthetic biology approach to minimal cells is pragmatic, though of unclear value to evolutionary questions pertaining to cellular origins. As an engineering project it is very productive and has a straightforward definition: it is simply the microbe with the smallest genome that is able to grow in axenic culture. There may of course be numerous minimal genomes for growth media, and hence there need not be a definitive minimal genome (Smalley et al., 2003; Dewall and Cheng, 2011; Juhas et al., 2014). On the criterion of growth in axenic culture, an organism can be designated “free-living” if it can be cultured in the absence of other organisms. Through this criterion, *Mycoplasma genitalium* is a good candidate for a minimal genome (Hutchison et al., 1999), though growing it in this way has the effect of removing it completely from its natural context, where, as an obligate intracellular pathogen, it is not in the least bit free-living (Dewall and Cheng, 2011).

In contrast, the use of comparative genomics to reconstruct the hypothetical last universal common ancestor (LUCA) has been less successful. A range of studies indicate that few genes are common to all three domains of life (Bacteria, Archaea, and Eukaryotes), and fewer still can be said to have an evolutionary history consistent with placement in some hypothetical common ancestor (Harris et al., 2003; Koonin, 2003; Hoeppner et al., 2012; Goldman et al., 2013). Horizontal gene transfer (Koonin, 2003), secondary gene losses (Becerra et al., 1997), and loss of evolutionary signal (Penny and Poole, 1999; Penny and Zhong, 2014) all obscure or erase early evolutionary history. It is not clear how to establish which, if any, of the many processes for extracting a living from the environment is the most ancient (though opinions abound), and it seems there is little to be gained from revisiting this question with ever larger genomic datasets. Moreover, there is no reason to expect that the LUCA was in any way a minimal cell, and it is difficult to equate the two, other than to assess the core of processes common to known biological systems (Goldman et al., 2013). The common ground between these efforts is that both the LUCA and the minimal cell concept focus on the internal parts-list of the genome: the gene set.

The point of this piece is to begin thinking about early evolution against the backdrop of biological interactions. We think that this is helpful for several reasons. First, systems that exist in isolation are probably the exception rather than the rule. Second, evolutionary transitions theory has provided the means by which to understand the emergence of complex systems, from replicators to cells to eukaryote cells with organelles, and is prefaced on interactions.

As a way to navigate this topic, we briefly summarize three concepts:

1. The pragmatic minimal genome concept
2. The evolutionarily stable distributed genome
3. Coevolutionary ecosystem interactions

We will then consider how biological interactions may help inform our understanding of early evolution.

## The Pragmatic Minimal Genome Concept

As mentioned above, the pragmatic minimal genome concept has a straightforward definition. It is part of the wider question in biology of establishing, for any system, what is essential and what is functionally critical. “Essential” and “functionally critical” sound identical, but are not. In transposon mutagenesis studies, the definition of essential derives from whether a gene can be knocked out. For instance, two paralogs may each be knocked out individually, such that global transposon mutagenesis (Hutchison et al., 1999) and related Tn-seq methods (Barquist et al., 2013) would designate each a non-essential gene. However, if knocking out both is lethal, then having at least one of these genes is functionally critical: without one of these genes, the function is not maintained. Hence the process is functionally critical, but the individual genes are not essential. It is this combinatorics problem (establishing how many genes can be knocked out simultaneously) that makes generating, rather than inferring, a minimal genome such a challenge.

From the point of view of a minimal cellular system, growth in axenic culture focuses enquiry on individual cells, in the context of a controlled environment. However, it has been pointed out that some model systems are neither naturally free-living nor autotrophic, so are clearly dependent on other organisms (Dewall and Cheng, 2011). In this view, free-living autotrophs would constitute a more appropriate system, because they can grow on a minimal medium composed of simple compounds that may have an abiotic origin. That said, it is clear that organismal and ecological interactions are not absent from candidate minimal species such as *Prochlorococcus* (Coleman and Chisholm, 2007; Lindell et al., 2007). Indeed, *Prochlorococcus* lacks catalase and has been shown to be dependent upon the hydrogen peroxide scavenging capacity of other microbes (Morris et al., 2011). More generally, genomic streamlining may drive loss of expensive, leaky traits as these can be derived through interactions (Morris, 2015). There may well be cellular entities that have few or no interactions with other biological systems—perhaps in deep subsurface communities where chemolithoautotrophs derive resources directly from mineral sources, there are few interactions because cell densities are low. A recent study estimates <1 cell/gram of sediment in samples from 2.5 km below the sea floor (Inagaki et al., 2015). Such extremes aside, it seems likely that most microbes do not exist in splendid isolation.

Against this backdrop of interactions, that genomes smaller than that of *M. genitalium* derive from bacterial endosymbionts indicates that, while genomes with fewer genes do exist, they do so not in isolation, but in close interaction. In comparison to the inferred minimal genome of *M. genitalium*, which carries an estimated 382 genes (Glass et al., 2006), the genome sequence of *Carsonella*, an endosymbiont of psyllids, is both smaller and carries fewer genes (160 kb, 182 open reading frames (ORFs); Nakabachi et al., 2006). However in this instance, it seems that this endosymbiont has too few genes to perform all the processes required for independent reproduction. Thus, endosymbionts such as *Carsonella* fall outside the pragmatic minimal genome concept. That said, if we ignore the biological context required for reproduction, replicating entities can carry much less genetic information than *Carsonella*. The logical, though absurd, endpoint is a minimal replicating element (Wegrzyn, 2001), which at its extreme is a single nucleotide [see (Dawkins, 1982) who noted this and labeled it “reductio ad absurdum,” and Griffiths and coworkers for subsequent discussion (Griffiths and Neumann-Held, 1999; Sterelny and Griffiths, 1999)].

## The Evolutionarily Stable Distributed Genome

While the genome of *Carsonella* is a stunning example of a highly-reduced endosymbiont genome, the context of the endosymbiont is clearly key—it is only possible to understand the genome within the context of coevolution with its host. In a number of cases, it seems that the host and endosymbiont have become so tightly integrated that one cannot exist without the other.

Indeed, endosymbionts like *Carsonella* and *Buchnera*, the maternally-inherited obligate endosymbiont of aphids, may well be on their way to becoming organelles, owing to ongoing genome reduction (Andersson, 2000, 2006). Taken in this light, their genomes are much more intact than genomes resulting from much older endosymbiotic interactions, such as the mitochondrion and chloroplast, and diminutive nucleomorph genomes resulting from secondary endosymbiosis (Archibald, 2007). The logical genomic endpoint of reductive evolution is an organelle without genes. This is seen in hydrogenosomes, the majority of which now completely lack DNA [though some carry genomes larger than the human mitochondrial genome (de Graaf et al., 2011)]. In this instance, the process of genome integration is complete, as all genes have relocated to a single compartment (the nucleus). In most cases, this endpoint may not be possible: the redox regulation of gene expression may necessitate the retention of genes in both chloroplasts and mitochondria (Allen, 2015).

Thus, some endosymbiont genomes clearly derive from free-living lineages, but may be difficult or impossible to grow in axenic culture, as they are far too dependent upon their host. In these instances, they may be well on the way to becoming part of a distributed genome. By this, we mean that the set of essential genes are distributed across multiple genomes in the same cell (perhaps organism) and coevolve and operate as a single evolutionary unit. In this respect distributed genomes are an evolutionarily stable state deriving from what once were individual entities (Maynard Smith, 1991; Kiers and West, 2015; Szathmáry, 2015). The “minimal number” of genes retained in one compartment is clearly less informative than this broader evolutionary understanding of the process.

More generally, the distinction between the state immediately prior to the evolutionary transition and directly after may be difficult to assess, and, in terms of genetic events, a bit of a holy grail: it may be futile to try to state which genetic change—a specific instance of gene loss, compartmental transfer, or coevolutionary molecular interaction—was the point at which the transition occurred. There is nothing to say transitions require one specific discrete change, though, viewed as a spectrum from pre-transition to post-transition, there are states that are clearly one or the other. Considered as a process, whether there is an exact event becomes less important.

One final point requires us to return to the example of *Mycoplasma*, where, because it is an obligate intracellular parasite, there is an asymmetry to the dependency: the parasite can become highly specialized and dependent upon its host, while the host would be quite happy in the absence of the parasite, even if it may have coevolved in its presence. Note that this kind of “extended genome” is not an evolutionary unit, so does not fit the above definition of a distributed genome.

## The Isolated Minimal Genome Versus Coevolutionary Systems

Distributed genomes are the product of an evolutionary transition, where none of the parties can revert to the ancestral, unintegrated state. Distributed genomes have clearly emerged in the evolution of the eukaryote cell (Szathmáry, 2015), and in secondary endosymbiosis (Curtis et al., 2012). This process has clearly been repeated multiple times in eukaryote evolution, and interactions between eukaryote hosts and their bacterial endosymbionts may represent early stages in this process (Poole and Penny, 2007). Thus, endosymbionts that are well on the spectrum to becoming organelles are not minimal genomes, but may be part of an emerging distributed genome, though it may be difficult in practice to determine the tipping point at which a set of closely interacting individuals becomes an evolutionarily stable, distributed genome.

As mentioned earlier, it seems difficult to imagine many biological systems that exist in total isolation. In that respect, most biological systems involve genome interactions at some level, and many of the species with the most minimal genomes show extensive genome-level dependencies. Consider the following thought experiment:

*“Try to imagine a plant that can survive and reproduce in a real ecosystem without using, in addition to its nuclear genome, most of the following: a mitochondrial genome (to convert energy); a chloroplast genome (to regulate photosynthesis); one or more mycorrhizal fungal genomes (to improve nutrient and water uptake); the genomes of pollinators (to assist in reproduction); and the genomes of a few birds, mammals, or ants (to move seeds around the ecosystem).”* (Thompson, 2006)

This illustration at once indicates that few, if any, genomes exist in genuine isolation from any other genome. In that respect, it may be tempting to state that the pragmatic minimal genome, by only requiring growth in axenic culture, utterly divorces the genome from its biology. In one respect, that is certainly the case: it is an extreme that follows from isolating the organism from its lifestyle, and which focuses on a very different research question than the ecological one raised above. However, this ecological idea could conceivably be taken to another extreme—that all life is a globally-distributed genome. Indeed, there has been some debate along these lines in ecological circles (Dagg, 2002, 2003). While few would subscribe to a “genomic Gaia,” this point is relevant because this type of model has been mooted in models of early cellular life, where the ideas of a planetary megaorganism (Mathieu and Sonea, 1995) or communal ancestor driven by global horizontal gene transfer (Woese, 2002; Goldenfeld and Woese, 2007) have become popular (Kim and Caetano-Anolles, 2011). There is every reason to expect horizontal gene transfer to have played an important role in early evolution, where it may have contributed to the emergence of the modern genetic code (Vetsigian et al., 2006). However, the difficulty for both some sort of genomic Gaia and microbial planetary megaorganisms is that they both rely on strong group selection and ignore the effect of selection at lower levels (Dawkins, 1982; Williams, 1992; Dagg, 2003; Poole, 2009; Szathmáry, 2015).

Difficulties with the communal ancestor model in light of levels of selection has been discussed in detail elsewhere (Poole, 2009), but one general difficulty with this class of model is that it is susceptible to parasites. Indeed, some simple systems such as hypercycles (where each gene in the cycle replicates itself and the next gene in the cycle) can be crashed by a parasite (an element that prefers to replicate instead of the next gene in the cycle; Maynard Smith, 1979). Such cycles are stable where there are barriers to interaction (Boerlijst and Hogeweg, 1991), which moves us away from a communal ancestor, where transfer barriers do not exist and are in fact selected against (Woese, 1998). Moreover, cellular membranes would in themselves represent a natural barrier to gene transfer (thus requiring the evolution of active transfer processes; Poole, 2009), suggesting a system where gene transfer was dominant and vertical inheritance insignificant would be difficult to explain on biophysical grounds. Finally, mobile elements that spread effectively through horizontal gene transfer have evolved to exclude competitors. A good example of these are toxin-antitoxin systems, which, when coded on a plasmid, will exclude other elements via a process of post-segregational killing (Cooper and Heinemann, 2000, 2005). In this process, competitor plasmids cannot invade the population because loss of the incumbent—toxin-antitoxin bearing—plasmid leads to cell death (because the toxin is longer-lived than the antitoxin, so loss of both genes leads to toxin-induced death). As a result, any displacement events are ultimately futile. One class of toxin-antitoxin system that has a broader effect on transfer are restriction-modification systems, which consist of a modification methylase, that marks DNA at specific palindromic motifs, and a restriction endonuclease, which cleaves at those same motifs, but only if they are not methylated. This leads to the cleavage of any DNA that comes into the cell from a foreign source (viruses, plasmids and cells lacking the restriction-modification system; Naito et al., 1995, 1998), so is a form of molecular patch-protection by a horizontally-transmitted parasite. The net effect is thus that all transfer events are reduced. Thus, we conclude that all three of these effects (unhindered parasitic spread, biophysical barriers, and reduction in transfer levels by horizontal replicators) all speak against a communal ancestor model.

In summary, the simple answer to why a globally-distributed genome is not viable in the forms presented thus far is that they confuse interactions between entities (be they mobile genes interacting via co-occupancy of compartments or organisms interacting in an ecosystem) and levels of selection (Poole, 2009). The theoretical tools to resolve this are well developed—this is the theory of evolutionary transitions (Szathmáry, 2015).

## Implications for Early Evolution

It is our view that the pragmatic approach to minimal genomes works completely adequately for synthetic biology. Though the combinatoric problem of shaving genes from the parts list may be difficult in practice, an approximate answer can be arrived at and incrementally improved. A completely different approach is building a cell completely from scratch, as per Gánti’s chemoton model (Gánti, 2003). Though at present a theoretical conception, it has value for both origins and as an alternative approach to the genome design question owing to its generalization of the problem. The area where we think that the minimal genome concept is less pragmatic, and more in need of discussion, is with regard to early evolution. We therefore end with a brief consideration of the three concepts described in this paper: pragmatic minimal, distributed genome, and coevolutionary interaction.

In the case of the pragmatic minimal genome, the genome is studied in complete isolation. Under a model where cells emerge from an abiotic environment, minimal cellular systems would be autotrophs of some kind with a minimal set of environmental interactions. This is possible, but ignores how cells evolved in the first place. It is clear that early pre-cellular systems are the product of gene-level interactions. The emergence of entities such as chromosomes and cells are still best understood in terms of evolutionary transitions following on from interactions between individual entities (Szathmáry, 2015), and there is emerging experimental support for cooperative behavior as a feature of RNA-based systems (Vaidya et al., 2012; Higgs and Lehman, 2015). Second, it suggests that our view of LUCA has been far too skewed toward a parts-list, and removes the cell from its surrounds. In that regard, the ideas of Forterre, who has championed coevolutionary interactions between viruses and cells as an important feature of early evolution (Forterre, 2006; Forterre and Prangishvili, 2009), help us in appreciating that cells probably never existed in isolation. As nicely explained by Gogarten and colleagues (Fullmer et al., 2015), both Black Queen evolution, where interdependencies emerge following loss from some individuals of a leaky common good gene (it is leaky because producers cannot monopolize the product), and horizontal transfer of pan-genome genes between individuals both involve coevolutionary interactions.

We also need to be careful not to overextend the reach of biological interactions. As discussed above, this has been done repeatedly in other areas of biology, mostly because of a misunderstanding of which levels selection operates. In this regard, a communal ancestor or megaorganism model is problematic because, from the perspective of levels of selection, the megaorganism is no different from the unworkable Gaia hypothesis. In its form of a single, interconnected communal system, it can only work if there are multiple communal ancestors interacting and competing for a common resource. This point is no different from the point Dawkins made in regard to Gaia requiring earth-like planets in competition for the same resource (Dawkins, 1982). The important point here is that, theoretically, both can work—Gaia works if multiple planets are competing(!), and a communal ancestor/megaorganism only if multiple separate communal ancestors are in direct competition. That seems difficult, and, importantly, is unnecessary, if the main point of the communal ancestor is to highlight the potential for horizontal gene transfer to contribute to early evolution: it is possible to have gene transfer without invoking this extreme. Thus, the lesson of past errors of interpretation is clear: in refocusing on interactions, we need to keep levels of selection front and centre.

Thus, in the world before eukaryote cells, which were the first entities with distributed genomes (an evolutionary transition), interactions between cells were effectively a set of ecological interactions (akin to the mycorrhizal fungi, pollinators and seed dispersers in the scenario quoted from Thompson, above). The real difficulty is that metabolic interactions between early cells are completely invisible to us. It may therefore be most productive to study processes such as the emergence and evolutionary stability of ecological interactions (Foster and Wenseleers, 2006; Morris et al., 2014; Oliveira et al., 2014; Morris, 2015) rather than searching for historical interactions. In that regard, it is worth noting that simple ecological interactions can be described as hypercycles (Maynard Smith and Szathmáry, 1995). More practically, minimal ecosystems (Guerrero et al., 2002), modeling (Oliveira et al., 2014), and experimental study of Black Queen evolution (Morris et al., 2014) and syntrophic interactions (Mee et al., 2014) all suggest a productive way forward for the study of the evolution of early cellular life, as these place both genome and organism in a biological context.

### Conflict of Interest Statement

The authors declare that the research was conducted in the absence of any commercial or financial relationships that could be construed as a potential conflict of interest.
